# Analysis of Transmission of MRSA and ESBL-E among Pigs and Farm Personnel

**DOI:** 10.1371/journal.pone.0138173

**Published:** 2015-09-30

**Authors:** Ricarda Maria Schmithausen, Sophia Veronika Schulze-Geisthoevel, Franziska Stemmer, Mohamed El-Jade, Marion Reif, Sylvia Hack, Alina Meilaender, Gabriele Montabauer, Rolf Fimmers, Marijo Parcina, Achim Hoerauf, Martin Exner, Brigitte Petersen, Gabriele Bierbaum, Isabelle Bekeredjian-Ding

**Affiliations:** 1 Institute of Medical Microbiology, Immunology and Parasitology, University Hospital Bonn, Bonn, Germany; 2 Institute of Animal Science, Preventive Health Management Group, University of Bonn, Katzenburgweg 7–9, Bonn, Germany; 3 Institute of Medical Biometry, Epidemiology and Computer Science, University Hospital Bonn, Bonn, Germany; 4 Institute of Hygiene and Public Health, University Hospital Bonn, Bonn, Germany; 5 Division of EU cooperation/ Microbiology, Paul-Ehrlich-Institute, Langen, Germany; Kent State University, UNITED STATES

## Abstract

Livestock-associated bacteria with resistance to two or more antibiotic drug classes have heightened our awareness for the consequences of antibiotic consumption and spread of resistant bacterial strains in the veterinary field. In this study we assessed the prevalence of concomitant colonization with livestock-associated methicillin-resistant *Staphylococcus aureus* (LA-MRSA) and enterobacteriaceae expressing extended-spectrum betalactamases (ESBL-E) in farms at the German-Dutch border region. Nasal colonization of pigs with MRSA (113/547 (20.7%)) was less frequent than rectal colonization with ESBL-E (163/540 (30.2%)). On the individual farm level MRSA correlated with ESBL-E recovery. The data further provide information on prevalence at different stages of pig production, including abattoirs, as well as in air samples and humans living and working on the farms. Notably, MRSA was detected in stable air samples of 34 out of 35 pig farms, highlighting air as an important MRSA transmission reservoir. The majority of MRSA isolates, including those from humans, displayed tetracycline resistance and *spa* types t011 and t034 characteristic for LA-MRSA, demonstrating transmission from pigs to humans. ESBL-E positive air samples were detected on 6 out of 35 farms but no pig-to-human transmission was found. Detection of ESBL-E, e.g. mostly *Escherichia coli* with CTX-M-type ESBL, was limited to these six farms. Molecular typing revealed transmission of ESBL-E within the pig compartments; however, related strains were also found on unrelated farms. Although our data suggest that acquisition of MRSA and ESBL-E might occur among pigs in the abattoirs, MRSA and ESBL-E were not detected on the carcasses. Altogether, our data define stable air (MRSA), pig compartments (ESBL-E) and abattoir waiting areas (MRSA and ESBL-E) as major hot spots for transmission of MRSA and/or ESBL-E along the pig production chain.

## Introduction

The use of antibiotics for therapy and growth promotion (not allowed in E.U. [1]) has led to the selection of antibiotic resistant bacteria and spread of antibiotic resistance genes [2–5]. Antibiotic resistant bacteria and their resistance determinants in livestock are not restricted to animals: Firstly, with glycopeptide resistance as a prominent example, we have observed that resistance genes can make their way into bacterial species that are more virulent for humans than those where the resistance was first observed [6–8]. Secondly, with the increasing prevalence of livestock-associated methicillin resistant *Staphylococcus aureus* strains (LA-MRSA) we are experiencing the spread of livestock associated resistant pathogens to humans [9–14].

Despite all achievements in hygiene and technology one of the major challenges in health care in developed countries is the prevention and treatment of nosocomial infections. The major threat is the silent spread of colonizing multidrug resistant pathogens among patients with overt risk for acquisition of resistant bacteria and—even worse—into those with no history of hospitalization or travel [15, 16]. These colonizers represent the major source for endogenous infections that occur after surgery, chemotherapy or other medical treatments associated with transient or prolonged immune suppression. Although multidrug resistance is presently defined as resistance to three or more classes of antibiotics [17], it should be noted that any lack of therapeutic effectiveness due to resistance to the administered substance can be devastating. The main dangers associated with these infections are aggravation of disease due to unexpected ineffectiveness of antibiotic therapy in a severely ill patient and the uncontrolled spread of these organisms in the hospital environment.

In views of these consequences the landscape within the research field dealing with bacterial resistance has changed. It has become evident that apart from describing the genetically based resistance mechanisms it is additionally necessary to study the origins and habitats of resistant bacteria. This is especially important because multidrug resistance does not only imply the acquisition of genes mediating resistance against different classes of antibiotics but is also associated with resistance to bacteriotoxic environmental conditions such as exposition towards heavy metals or disinfectants [18–20]. This trend has also fostered research in the agricultural field, which addresses the consequences of antibiotic consumption in the veterinary field, including the assessment of the potential role of livestock as a reservoir for transmission of multidrug resistant bacteria to the human host [3, 4, 21].

Methicillin-resistant *Staphylococcus aureus* (MRSA) is one of the most widely studied resistant bacterial species in this context. Epidemiologically discernable livestock-associated (LA-) MRSA strains have evolved next to the community acquired (CA-) and hospital acquired (HA-) MRSA lineages. The LA-MRSA strains have particularly adapted to pigs as hosts [22] and have been detected at all different levels of the pig production chain [23–25]. Notably, LA-MRSA strains have been isolated from persons who are in close contact with pigs and they are more frequently detected in hospitals within rural areas [26–28].

More recent work has described the emergence of enterobacteriaceae resistant to betalactam antibiotics expressing extended beta lactamases (ESBL-E) in pigs [29]. ESBL-E frequencies among patients have increased worldwide. This has propagated the broad use of betalactamase inhibitors and further selection of highly resistant strains [30, 31]. When combined with quinolone resistance, ESBL expression poses a serious clinical problem due to limited options for oral antibiotic therapy, which make intravenous administration and hospitalization of the patient necessary. Evidently, the limited number of orally absorbed antibiotics available will also have important impact on antibiotic usage in pig farming. Furthermore, new hygiene measures are needed to prevent that colonized pigs or their meat turn into a new reservoir for transmission of ESBL-E to humans [32–36].

In the present study we assessed the prevalence of simultaneous MRSA and ESBL-E colonization throughout the pig production chain (from piglets to carcasses). These findings were related to concomitantly assessed MRSA and ESBL-E recovery in air samples and from humans living and working on farms.

## Material and Methods

### Study design and sampling approach

The primary aim of the study was to assess the prevalence of simultaneous colonization of pigs with MRSA and ESBL-E at different stages of pig production. The secondary goal was to correlate the prevalence with the presence of MRSA and ESBL-E samples from air and humans in the farm environment. The cross sectional study followed the pig production chain and was divided into two major parts: 1.) pig production and 2.) slaughtering process. Representative air samples and swabs from humans from the farm environment were collected in parallel. Details are provided in the specific sections below.

### Farms

Thirty-five pig farms (33 in North Rhine-Westphalia, Germany and 2 situated in the Netherlands) collaborating with two participating abattoirs (A+B) were enrolled in the study. Farms from all pig production steps were included and defined by categories [37]: farrowing (FR), nursery (NF) and finishing (FF). Farrowing farms belong to the breeding production stage. The farrowing sub-stage also includes the lactation of the young suckling piglets. The farrowing farms keep piglets up to 1–4 weeks (1.5–8 kg). The farrowing piglets are supplied to nursery farms for rearing the young piglet after weaning (newly weaned pig). Nursery farms raise piglets within the age of 4–12 weeks (8–30 kg) and provide nursery pigs to finishing farms. The finishing period is divided into an early (12–20 weeks, 30–50 kg) and a final finishing period (21–30 weeks, 50–120 kg). The finishing period marks the last step before slaughter. We covered the pig production chain from young farrowing piglet (with no investigation of sows) to the carcasses including the following stages: young farrowing piglet, farrowing piglet, newly weaned pig, nursery pig, early finishing pig, finishing pig, carcass. The transfer of bacteria from sows to piglets was not determined. Based on voluntary participation we were able to include 10 FR, 2 NF and 23 FF.

### Participation of farms

The participating farms were recruited for participation in the hygiene monitoring program by their pig producer association. The hygiene monitoring program was an initiative of the pig producer association in collaboration with the agricultural faculty of the University of Bonn. Farms with ≤ 2 pig suppliers were selected for participation. The samples were collected on the farms but the individual pigs were not tracked to the next pig production level, with exception of the pigs transported and slaughtered at the abattoirs.

The farmers (owners) agreed with the collection of air samples and the sampling of the pigs on the farms. These samples were taken during routine sampling for monitoring and the sampling itself is non-invasive. According to the German animal welfare legislation this study was not an animal experiment. An approval by the regulatory body or an animal welfare committee was not necessary. Nevertheless, all measures taken strictly followed the terms set by the animal welfare committee of the University of Bonn. The data summarized in this study were part of a routine hygiene management monitoring program that was started to provide data on multidrug resistant bacterial colonization in pigs and farm employees and to control measures taken to reduce spread of resistant bacteria. No previous sampling on the colonization status with MRSA and/or ESBL-E had been performed. Therefore, no distinction could be made based on the MRSA and/or ESBL-E prevalence. No personal data were used or stored for the present study. Therefore, consent from the ethics committee was not required. The owners of the farms and the farm personnel were informed about the program and participated on a voluntary basis. In accordance with the declaration of Helsinki/Seoul written informed consent is available from all human subjects involved. The participating farmers provided the information on the antibiotic classes applied to the pigs that were sampled beginning from entry into the farm. This information was verified in their livestock protocols. The results of this study were communicated to the farmers.

### Sample collection in pigs

Sample collection was performed from June 2012 to September 2012. From all pigs included in the study we obtained a nasal swab (inserted into both anterior nares) for MRSA screening and an intrarectal swab for ESBL-E detection. Swabs with Amies medium and charcoal were purchased from MAST Diagnostica GmbH, Reinfeld, Germany. Five hundred fifty pigs were sampled; a total of 547 nasal swabs and 540 rectal swabs were analyzed; 3 nasal swabs and 10 rectal swabs did not reach the laboratory.

In the first part of the study, samples were obtained from two age groups housed in two different compartments per farm, i.e. the youngest and oldest age group per farm type: farrowing (young farrowing piglets: 1–2 weeks and farrowing piglets: 2–4 weeks)), nursery (newly weaned pigs: 4–6 weeks and nursery pigs: 9–12 weeks), finishing (early finishing pigs: 12–20 weeks, 30–50 kg and finishing pigs: 21–30 weeks, 50–120kg). Either 10 (farm B1-22) or 5 (farm B23-35) pigs per compartment were screened for MRSA and ESBL-E carriage. Farms were categorized by MRSA/ESBL-E frequencies as follows: Category A: MRSA / ESBL-E free; Category B: > 0 and ≤ 20%; Category C: > 20 and ≤ 50%; Category D: > 50% MRSA/ESBL-E.

In the second part of the study the slaughtering process was subdivided into three sampling periods per pig, e.g. before transport to the abattoir (phase 1), immediately after slaughter (phase 2) and on carcasses in cold storage (phase 3). Transport time from farm to abattoir lay between 1–3 hours. After arrival pigs were separated into an own waiting area in abattoir A or the kept in the common waiting area (in abattoir B) before slaughtering. Thirteen farms selected from the first study period participated. To estimate the risk for contamination with MRSA and/or ESBL-E during the slaughtering process we collected samples from pigs from 7 finishing farms with absent to low (≤10%) MRSA and/or ESBL-E in the first sampling period and from 6 farms with higher frequencies (S6 Table). Samples were collected from three pigs per farm at three different time points: i.) on the farm, ii.) during slaughter (at 2 different abattoirs (A+B) and iii.) on the carcasses deposited in the cool room of abattoir A. At the abattoirs the specimen were taken as described for the farms. All areas defined for swab sampling of swine carcass surfaces according to ISO 17604:2003/Amd.1:2009 were sampled with one swab per carcass. Discordant samples (positive on farm and negative in abattoir) were omitted (2 MRSA, 4 ESBL-E).

### Human specimen

Written informed consent was obtained from all participating human volunteers who live or work on the farms. All individuals were categorized as “contact” or “no contact” to pigs and tested for nasal carriage of MRSA and rectal carriage of ESBL-E. Nasal swabs for MRSA were taken from the vestibule of both nares by the responsible physician in the monitoring program; ESBL-E screening was performed from fecal samples in fecal tubes (MAST Diagnostica GmbH), which were delivered by the individuals participating in the monitoring program.

### Air samples

The air collection was conducted using an MAS-100 NT® air sampler (Merck KGaA, Darmstadt, Germany). The air was suctioned through a perforated lid (300-x-0.6 mm openings) onto the surface of selective agar plates, e.g. CHROMagarMRSA^TM^ for MRSA (n = 70) or CHROMagarESBL^TM^ (MAST Diagnostica GmbH) and ESBL (n = 67), 30 sec or 10 minutes, respectively. The system used a mass air flow sensor for measuring the air inflow and to maintain the continuous regulation of the air intake volume during sampling. For the detection of ESBL-E, two measurements were performed with an air flow rate of 500 liters/min for five minutes per group. For detection of MRSA, we used an air flow rate of 100 liters/min for one minute (farm B1-22) or thirty seconds (farm B23-35 and abattoirs). The filter system was disinfected with alcohol pads after each measurement (B. Braun Melsungen AG, Melsungen, Germany).

On the farms, air samples were obtained from the center of the compartments 1.20 m above ground level with stable doors closed. At the abattoirs, air samples were collected in the waiting pen (abattoir A+B) and in the cold storage area (only abattoir A).

### Bacterial culture

All samples were stored at 4°C during transport to the laboratory. All specimen were inoculated within 48 hours. All swabs were streaked on Columbia / 5% sheep red blood agar plates (Becton Dickinson, Heidelberg, Germany) and selective agar plates, i.e. CHROMagarMRSA (MAST Diagnostica GmbH) for nasal swabs and CHROMagarESBL (MAST Diagnostica GmbH) for intrarectal swabs and feces. Plates were incubated at 37 ± 1°C for 24 h. Incubation of air sample plates was started on-site. Sealed plates were incubated for 48 h at 37 ± 1°C. The colonies were counted as total number of CFU/m^3^ and reported after statistical correction with the species-specific correction factor (Pr / r) according to Feller [38].

### Confirmation of MRSA and *spa* typing

After subculturing on Columbia sheep red blood agar, all presumptive *S*. *aureus* colonies were checked for hemolysis and confirmed by coagulase testing and MALDI-TOF MS (mass spectrometry) (VITEK MS, bioMérieux SA, Marcy l'Etoile, France). Antibiotic resistance was determined by agar diffusion tests (EUCAST criteria [39]) and MRSA confirmed by PBP2a Culture Colony Test (Alere Ltd, Stockport, UK). For each farm, one MRSA isolate per compartment (two per farm: young/old), two air and all human MRSA isolates were typed using *spa*-typing as described in [40] (148 MRSA isolates from farms and 48 MRSA isolates from abattoirs). Antibiotic resistance was tested by agar diffusion. For isolates with a zone diameter ≥ 16 mm, tetracycline resistance was confirmed by PCR detection of *tetM* and *tetK* [41].

### Identification, antimicrobial susceptibility testing and molecular typing of ESBL-E

All enterobactericeae detected on CHROMagarESBL were identified by MALDI-TOF MS. Antibiotic susceptibility was determined on VITEK-2 (bioMérieux SA) for all non-*E*. *coli* isolates, two *E*. *coli* ESBL-E per farm (one per compartment) and all *E*. *coli* ESBL-E isolates from air, humans and abattoirs. Results were interpreted by EUCAST criteria [39]. Presence of ESBL genes was confirmed by PCR. DNA was isolated using UltraClean Microbial DNA Isolation Kit (MO BIO Laboratories, Carlsbad, California, USA) and ESBL genes detected by the ESBL Assay from AID GmbH (Straßberg, Germany) using recombinant Taq DNA Polymerase (5U/μl, Thermo Fisher Scientific Inc., Waltham, Massachusetts, USA, #EP0401). AmpC and ESBL positive strains were further confirmed by AmpC&ESBL Detection Discs and Cefpodoxim ESBL ID Disc Set (both from MAST Diagnostica GmbH, Reinfeld, Germany) and ESBL E-Test (bioMérieux SA, Nuertingen, Germany). Molecular typing of *E*. *coli* strains was performed by repPCR using the Diversilab system (bioMérieux SA) [42]. One ESBL-E isolate per farm compartment (2 isolates per farm) and all ESBL-E isolated from air and abattoirs and humans were subjected to DiversiLab analysis. Pulsed field gel electrophoresis (PFGE) was carried out as previously described in [43]. In brief, SpeI (New England Biolabs, Marnes-La-Coquette, Frankreich) was used for enzymatic digestion of *E*. *coli* DNA in agar blocks and electrophoresis was performed on a Rotaphor®VI (Biometra GmbH, Göttingen, Germany) for 40 hours (50s log 5s,190 V,130 mA). Analysis was performed using the BioDocAnalyze (BDA) Gel Analysis BDA Software Version 2.66.3.44 9-990-015/English) Version 02/12.

### Statistical analysis

The Odds-ratio given for the simultaneous occurrence of ESBL-E and MRSA was replaced by Cochran-Mantel-Haenszel based relative risks estimates stratifying for farms. No regression models were used to estimate prevalences and their ratios. Acquisition of MRSA and ESBL-E in the abattoirs was calculated by McNemar test and the differences between abattoirs analyzed by Fisher´s exact test.

## Results

**Fig 1:**
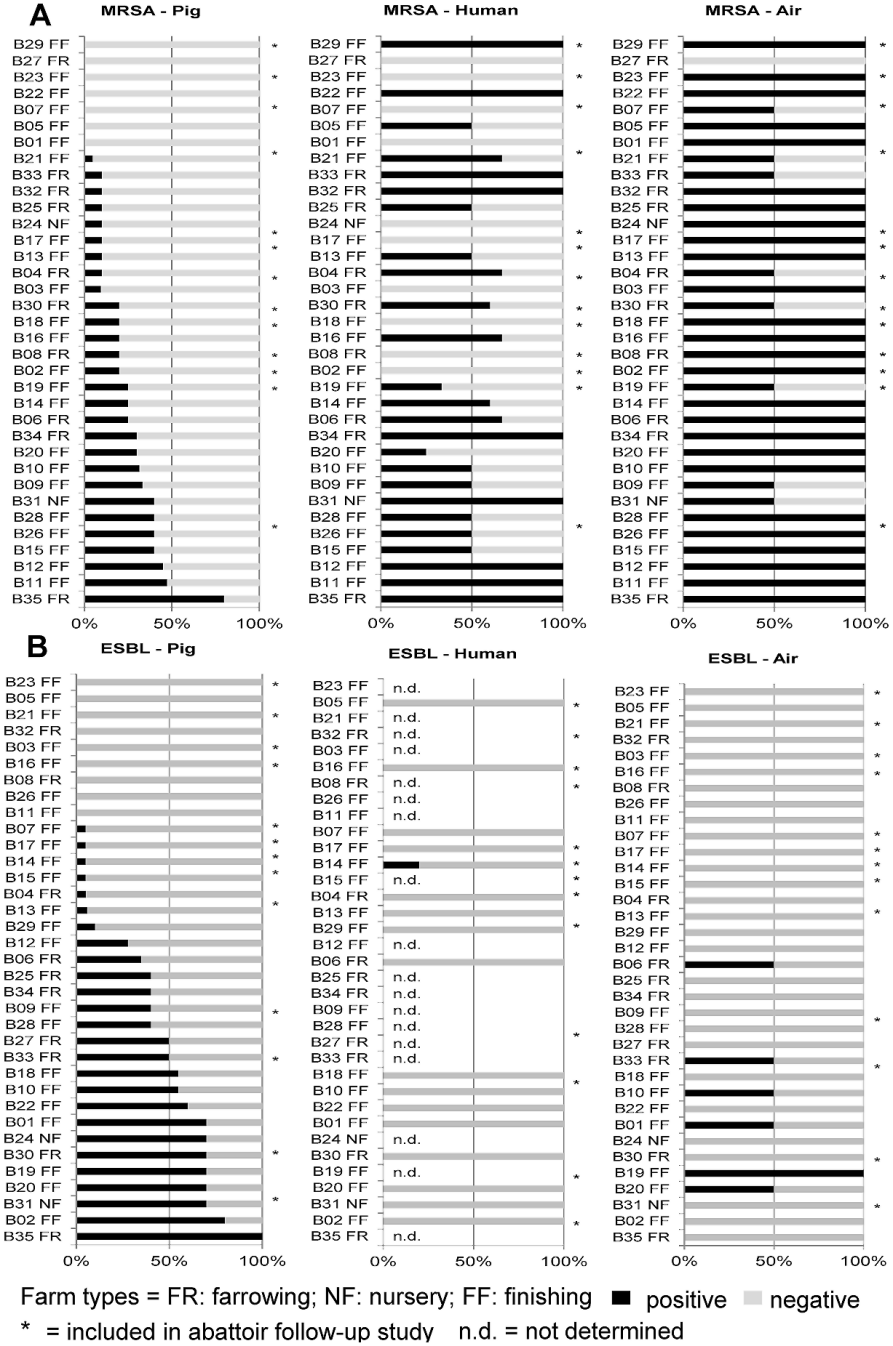
Comparative overview of MRSA and ESBL-E colonization in pigs, humans and air on farms. Samples from pig, human and air were collected on 35 pig farms (B#). Farm types (farrowing (FR), nursery (NF) and finishing (FF)) are provided in the diagram. All samples were analyzed for MRSA (**A**) and ESBL-E (**B**). The figure depicts the results obtained on the individual farms sorted by prevalence of MRSA (**A**) or ESBL-E (**B**), respectively. Bars depict the percentage of positive (black) and negative (gray) samples.

We analyzed the frequency of MRSA and ESBL-E in samples obtained from pigs from 35 farms located in the German-Dutch border region as well as two associated abattoirs, the respective farm environment (e.g. air) and persons living and working on these farms (see Fig 1 for overview).

### Prevalence of MRSA and ESBL-E in pigs

**Fig 2:**
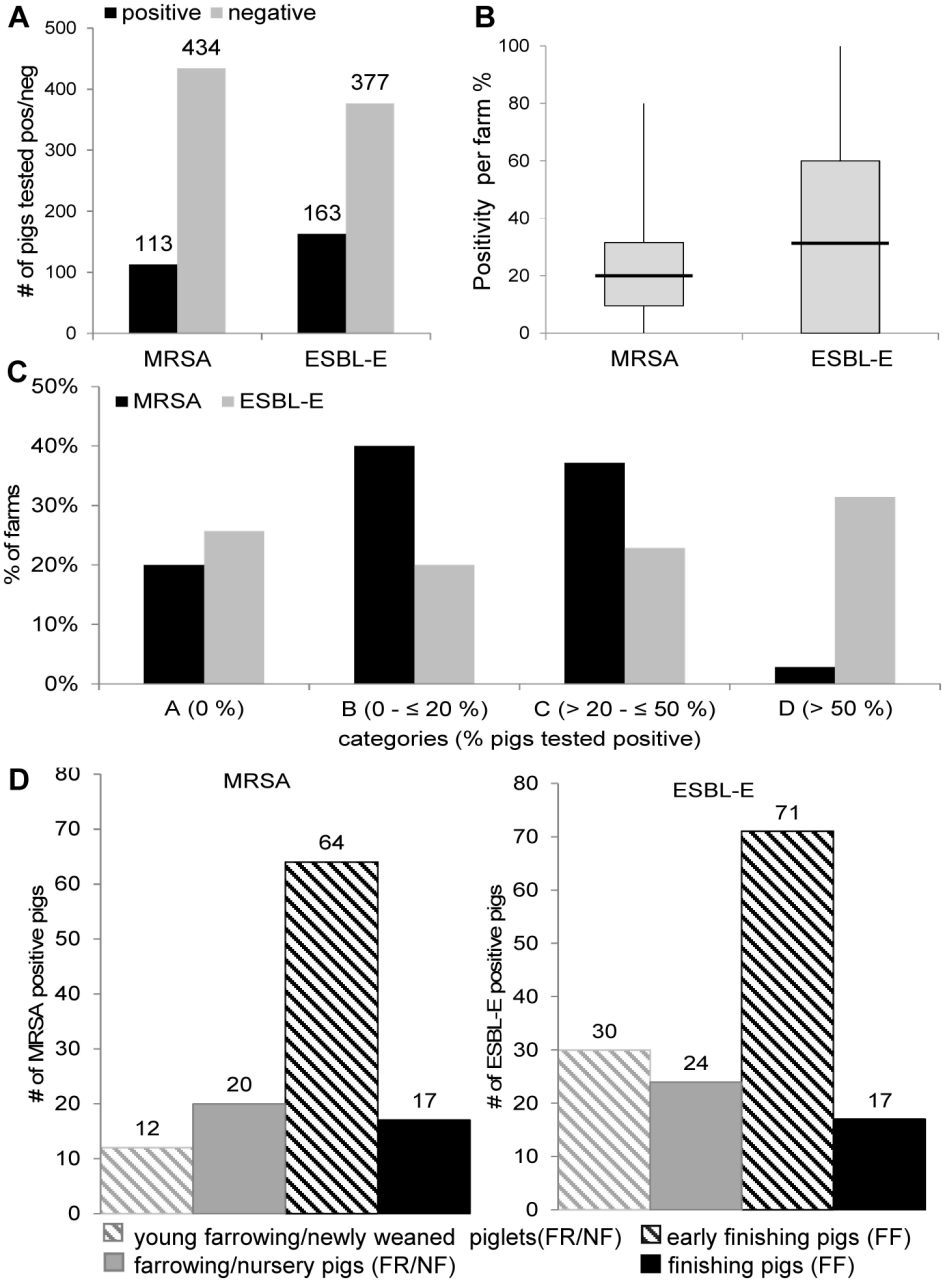
MRSA and ESBL-E recovery in pig samples. **A:** Summary of results obtained in all tested pigs. The graph depicts the absolute numbers of pigs tested positive (black bars) or negative (gray bars) for MRSA and ESBL-E. **B:** The graph depicts the range (boxplots) of colonization (in %) for MRSA (left) and ESBL-E (right) on farms. The median is indicated as a black line. **C:** Farms were categorized based on their MRSA (black bars) and ESBL-E (gray bars) colonization. Four categories (A to D) were defined as indicated in the diagram. **D:** Number of MRSA (left) and ESBL-E (right) positive pigs in different pig production steps and farm types (farrowing (FR) and nursery (NF) farms compared to finishing (FF) farms). Production steps within these farm types are categorized as young farrowing/newly weaned piglets (gray hatched bars) and farrowing/nursery pigs (gray bars) in FR/NF and early finishing (black hatched bars) and finishing pigs (black bars) in FF.

MRSA was detectable in 20.7% (113/547) of pigs and ESBL-E in 30.2% (163/547) of pigs (Fig 2A). Thus, total ESBL-E frequency was 32% higher than MRSA colonization. The majority of recovered ESBL-E were *Escherichia coli* isolates (155/163, 95.1%). Next to *E*. *coli* we detected seven *Citrobacter* spp. and one *Serratia fonticola* ESBL-E as well as two *Enterobacter cloacae* isolates, which were disregarded in the subsequent analyses. Double colonization with MRSA and ESBL-E was detected in (48/540) 8.9% pigs on 17 farms. This corresponds to (48/113) 42.5% of MRSA positive pigs and (48/163) 29.5% of ESBL-E colonized pigs. Overall, MRSA colonization correlated with ESBL-E recovery (p<0.001, RR (relative risk) = 2.37 CI (confidence interval) [1.70–3.55] to detect MRSA for ESBL-E positive pigs and RR = 1.69 [1.36–1.94] to detect ESBL-E for MRSA positive pigs).

### Frequency of MRSA and ESBL-E colonized farms

Analysis of MRSA and ESBL-E positivity on the farm level revealed that there were more farms with MRSA detection (80% (28/35) of farms) than farms with ESBL-E recovery (74.3% (26/35)) (Fig 1A and 1B). Two farms were free of MRSA and ESBL-E and 60.0% (21/35) had pigs colonized with both.

MRSA colonization ranged from 0–80% of tested pigs on a farm (median: 20%) (Fig 2B; S1 Table). As shown in Fig 2C, 20.0% (7/35) of farms were MRSA-free, e.g. Category A, 40% (14/35) Category B, 37.1% (13/35) Category C, 2.9% (1/35) Category D. By contrast, analysis of ESBL-E detection revealed that a higher percentage of farms was ESBL-E-free (25.7% (9/35); Category A) (Fig 2C). However, ESBL-E detection ranged from 0–100% (Median: 35%) (Fig 2B; S2 Table) and positivity within an affected farm was higher than with MRSA, e.g. 31.4% (11/35) of farms belonged to Category D, 22.9% (8/35) to Category C and only 20% (7/35) Category B.

### MRSA and ESBL-E colonization varies depending on the pig production level

In each farm we collected samples from two compartments, e.g. youngest and oldest pigs. In the farrowing and nursery step we defined the young pigs as “young farrowing/newly weaned” and the older pigs as “farrowing/nursery pigs”. In the finishing step we defined the young pigs as “early finishing” and the older ones as “finishing” pigs. Notably, MRSA and ESBL-E colonization frequency varied depending on the pig production level (Fig 2D, S3 and S4 Tables). Piglets with MRSA and ESBL-E colonization were found on both nursery farms (100%); on farrowing farms MRSA was present on 9/10 (90.0%) and ESBL-E on 8/10 (80.0%) farms. The overall prevalence of MRSA and ESBL-E isolated from pigs was lower on finishing farms, i.e. 73.9% (17/23) and 69.6% (16/23), respectively.

Further analyses revealed age-dependent differences in MRSA colonization frequencies (Fig 2D; S3 Table): in farrowing and nursery farms the MRSA positivity was 21.3% (32/150); 62.5% (20/32) of these MRSA were isolated from farrowing/nursery pigs and only 37.5% (12/32) were detected in young farrowing/newly weaned piglets. This was also reflected on a farm level, e.g. in 10/12 (83.3%) farms MRSA detection in farrowing/nursery pigs was ≥10% and only 2/12 (16.7%) were below 10%. Global MRSA frequencies in finishing farms were comparable to those in farrowing farms, e.g. 19.8% (81/410). On a farm level 16/23 (69.6%) of finishing farms had MRSA frequencies of ≥10% and only 7/23 (30.4%) were <10%. However, further analyses revealed a statistically significant difference between MRSA frequencies in early finishing pigs (79% (64/81)) compared to finishing pigs (20.9% (17/81)) (p < 0.001).

The overall ESBL-E frequency in farrowing and nursery farms was 36.0% (54/150). A higher prevalence was found in young farrowing/newly weaned piglets (55.6% (30/54)) compared to farrowing/nursery pigs (44.4%) (Fig 2D; S4 Table). On the farm level, ESBL positivity ≥10% was found in 10/12 (83.3%) “young farrowing/newly weaned” compartments and in 8/12 (66.7%) “farrowing/nursery” compartments. In finishing farms the ESBL-E positivity was lower, e.g. 26.6%. Early finishing pigs accounted for 65.1% (71/109) of positive ESBL-E and only 34.9% (38/109) were detected in finishing pigs. This difference was statistically significant (p < 0.0001). Analysis on a farm level showed that the ESBL-E detection in “early finishing” compartments was ≥10% on 14/23 finishing farms (60.9%). In “finishing” compartments 43.5% (10/23) farms displayed ESBL frequencies ≥10% and 56.5% (13/23) farms lay below 10%.

### MRSA and ESBL-E detection in humans working and living in the farm environment

48.8% (42/86) of samples from farmers, staff and family were tested positive for MRSA (S5 Table). On 21 farms MRSA was recovered on both pigs and humans. Of those tested positive only one person had no contact to pigs; in those individuals with no direct contact to pigs all but one person were MRSA negative (85.7%; 6/7). Persons who regularly came in contact with pigs were more frequently colonized with MRSA (53.2%; 42/79) when compared to those with no contact (14.3%; 1/7).

Fecal swabs from all individuals tested were positive for ESBL-E in 2.5% (1/40). The person with ESBL-E colonization was negative for MRSA but colonized with an MSSA (t005). This person had no contact to pigs but regular contact to the healthcare system. Altogether, colonization with ESBL-E was less frequent than that with MRSA.

### MRSA and ESBL-E transmission in the slaughtering process

We screened for MRSA and ESBL-E carriage in pigs before and after delivery to the abattoir and on the carcasses. Statistically significant acquisition of MRSA or ESBL-E in pigs tested negative before arrival at the abattoirs was observed in 29.7% (11/37) (p = 0.001) and 29.4% (10/34) (p ≤ 0.05), respectively (S6 Table). Nevertheless, neither MRSA nor ESBL-E were detected on the carcasses of the tested pigs. It is noteworthy that we observed differences in acquisition of MRSA and ESBL-E in pigs between abattoirs. The increases in MRSA and ESBL-E detection were as follows: abattoir A: MRSA 18.8% (3/16; not significant), ESBL-E 8.3% (1/12; not significant); abattoir B: MRSA 38.1% (8/21; p ≤ 0.001); ESBL-E 40.9% (9/22; p = 0.02). The difference between abattoirs was statistically significant for MRSA (p = 0.04). The handling of pigs before slaughter was different, i. e. there was a separated and dry waiting area in abattoir A, while pigs were randomly mixed and irrigated in abattoir B.

### Evaluation of air as transmission medium

To correlate MRSA and ESBL-E content in the stable air with pig and human colonization we analyzed air samples collected by impaction (S7 Table). Samples were obtained in all stable compartments where pigs were sampled. MRSA were detected in the stable air of 34 out of 35 (97.1%) pig farms tested (i. e. ≥1 out of 2 samples positive). On 74.3% (26/35) of farms MRSA were detected in air samples from both compartments and in 22.9% (8/35) of farms only one compartment (young/old) was contaminated. There was no difference between farrowing/nursery and finishing farms.

Notably, air samples from one farm were completely free of MRSA (B27). This farm was also classified as Category A in pig sampling. On three farms MRSA was detected in the air but was absent in the samples obtained from pigs and humans.

ESBL-E positive air samples were found on 17.1% (6/35) of investigated pig farms. All of these farms were affected by both ESBL-E and MRSA colonization in pigs (ESBL-E Categories: C (1 farm), D (5 farms) and MRSA Categories A (1 farm), B (1 farm), C (4 farms)). Comparative analysis of farrowing/nursery versus finishing farms displayed no relevant difference in air positivity related to the pig production level, e.g. 16.7% (2/12) and 17.4% (4/23), respectively.

Air sampling in the abattoirs delivered the following results: the MRSA frequency was comparable to that in the air samples obtained on farms, e.g. 13/14 (92.9%). However, the ESBL-E frequency was higher than on farms, e.g. 6/12 (50.0%). Due to the low sample numbers obtained in the abattoirs a statistical comparison of farms and abattoirs was statistically not appropriate; the trend, however, was clear. Notably, no relevant differences were found in the comparison of abattoirs (S8 Table). Collectively these data indicate that MRSA contamination of air is more wide-spread than for ESBL-E. However, there was no correlation of ESBL-E detection in air with that in humans.

### Antimicrobial susceptibility testing of MRSA isolates

LA-MRSA strains belong to the ST398 lineage and are characterized by tetracycline resistance [9, 44, 45]. All 196 strains tested were resistant to penicillin and cefoxitin. By agar diffusion testing 191/196 (97.4%) of isolates were further resistant to doxycycline, a characteristic of LA-MRSA. Tetracycline resistance in isolates with zone diameters ≥16mm was confirmed by detection of *tetM* (2 isolates) or *tetK* (1 isolate) resistance genes by PCR.

### Molecular typing of MRSA

**Fig 3:**
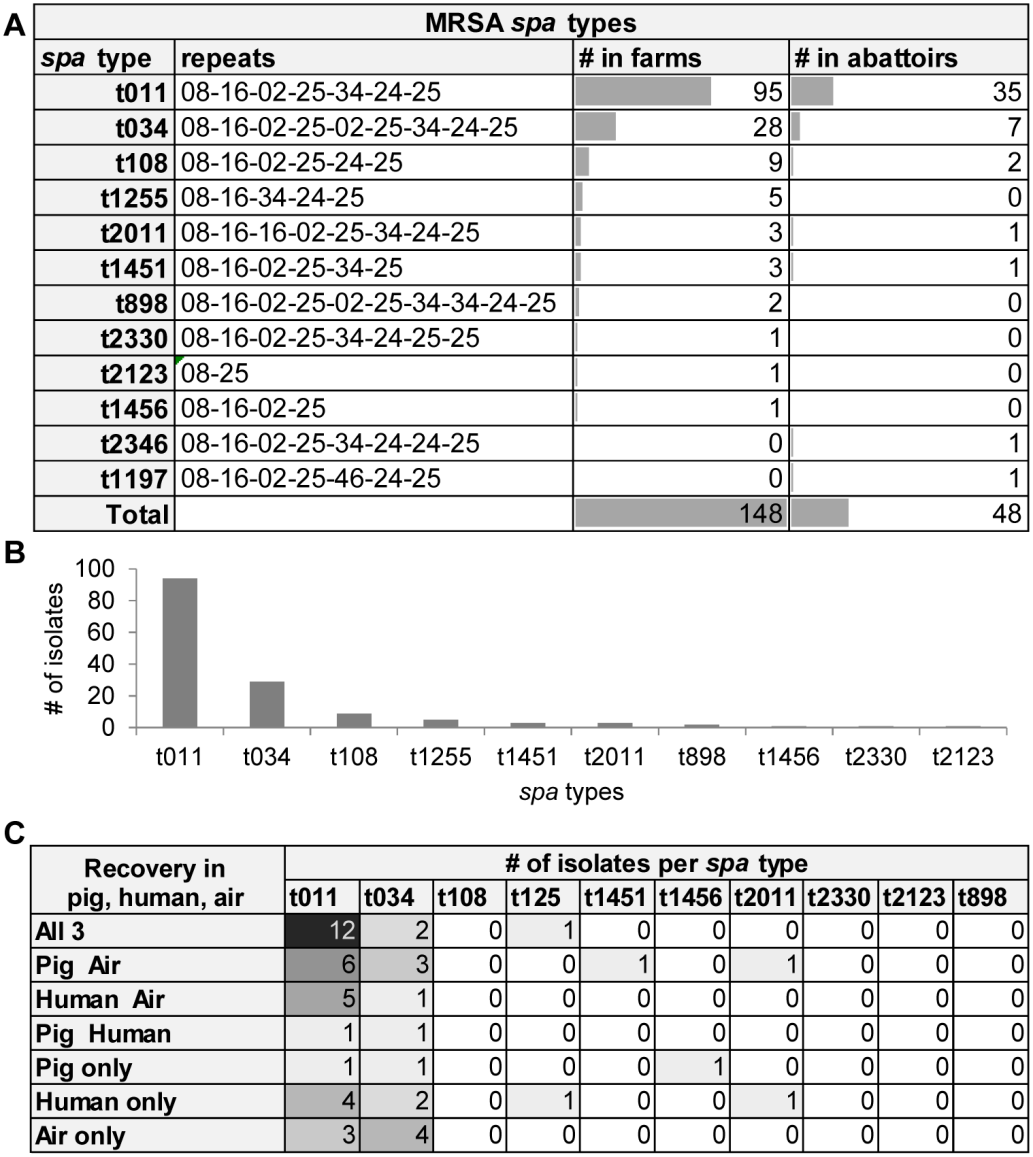
*spa* typing of MRSA isolates. **A:** *spa* types of MRSA isolates obtained from pigs, human and air. **B:** Prevalence of *spa* types detected on farms and on abattoirs. **C:** Analysis of *spa* types in regard to their simultaneous presence in different media (pig, human, air).

To confirm the LA-MRSA lineage of the isolates we performed *spa* typing (Fig 3A). The *spa* types most frequently isolated from snouts and air were t011 (n = 130) and t034 (n = 35) (Fig 3B; S9 Table). All MRSA *spa* types belonged to the ST398 lineage. Only two MSSA isolates recovered from human nasal swabs corresponded to *spa* types t005 and t491 that do not belong to this lineage.

Notably, all MRSA isolates found in humans, pigs and air were confirmed to be LA-MRSA by *spa* typing. The *spa* type t011 was found in 32/35 participating farms (91.4%); t034 was detected on 20/35 farms (57.1%) and t108 on 6/35 farms (17.1%). In 12 out of 35 farms (34.3%) t011 was found in pigs, humans and air (Fig 3C). This was also observed with t034 (B04, B09) and t1255 (B30) (S9 Table). In additional 6 out of 35 farms (17.1%) t011 was detected in humans and air but not in pigs. No relevant differences in *spa* type distribution were found between abattoir and farm and between air, pigs and humans. However, *spa* types t2346 and t1197 were only recovered from abattoir B.

### Analysis of ESBL enzymes reveals predominance of CTX-M

The presence of ESBL genes was confirmed by PCR analysis. We detected ESBL enzymes in 69 of 72 third generation cephalosporin resistant *E*. *coli* strains, thus proving the high specificity of the medium used for ESBL-E selection [46]; two isolates were AmpC positive. The majority of *E*.*coli* ESBL-E isolates, i.e. 95.7%, were CTX-M positive as reported previously in [33, 47–52]. One isolate harbored CTX-M and TEM AS 238 S, another contained SHV AS 238/240 and a third isolate harbored TEM AS 238 S and TEM AS 104 K.

### Rep-PCR-typing of ESBL-E isolates reveals heterogeneity of ESBL-E

To assess whether the *E*. *coli* ESBL-E strains isolated arose from a common strain we performed a repetitive element PCR (rep-PCR) analysis of the purified DNA samples using the DiversiLab system. This method offers a rapid and automated method for genotyping with high reproducibility and the important advantage of an electronic database. Cut-off values were set at 98% similarity to increase the discriminatory power of the method [43, 53, 54].

**Fig 4:**
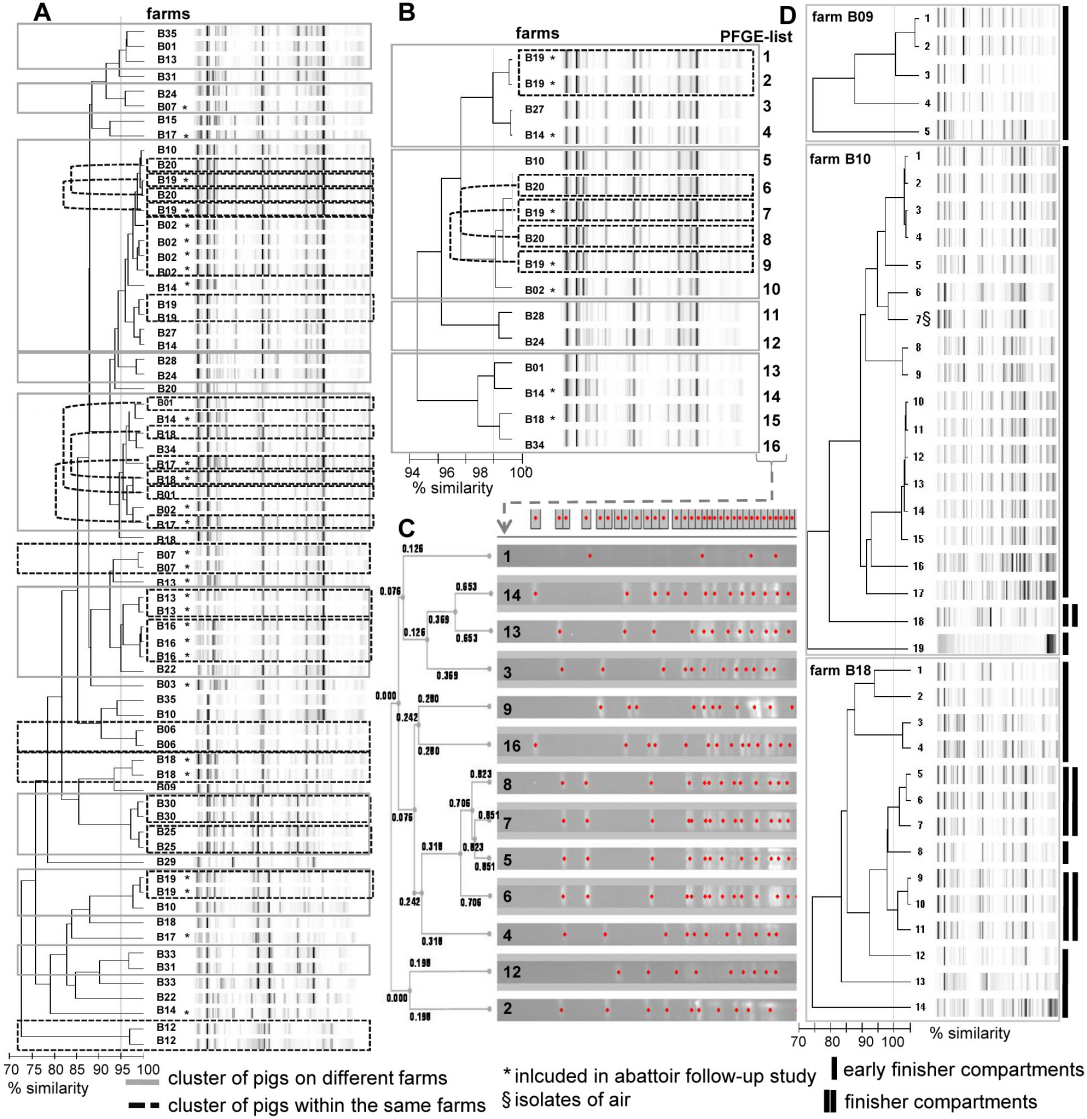
Molecular typing of *E*. *coli* ESBL-E isolates. Representative ESBL-E isolates from pigs, air and human were analyzed by repPCR (Diversilab, Biomerieux, Nürtingen, Germany) (**A**, **B** and **D**) or pulsed field gel electrophoresis (PFGE) (**C**). **A, B and D:** Diversilab typing results. Clusters of isolates obtained from different farms are marked by gray rectangles and clusters of isolates from pigs derived from the same farm are marked by black hatched lines. **A:** Overview of Diversilab typing results of representative ESBL-E isolates. The cut-off value was set at 95% similarity. **B and C:** To confirm strain relatedness all ESBL-E isolates from clusters with a similarity of ≥ 98% (summarized in **(B)**) were reanalyzed by pulsed field gel electrophoresis (PFGE) **(C)**. **D:** On three exemplary farms with high ESBL-E prevalence (B09, B10, B18) all ESBL-E isolates from the same farm were subjected to Diversilab analysis to test for strain relatedness within the farm and/or a single compartment. Isolates from early finishing compartments are marked by black lines and those from finishing compartments by double black lines. Isolates from air are indicated by a “§”.

The results obtained revealed genetic heterogeneity of strains among the different farms (Fig 4A). However, a few clusters with high similarity (≥98%) composed of isolates from different farms were also detected (Fig 4B). The isolates within these clusters were subjected to PFGE analysis, which confirmed strain relatedness in some but not all cases. The results are shown in Fig 4C.

Notably, strain relatedness was found in simultaneously collected isolates from air and pigs (Fig 4A) but isolates obtained at the abattoirs did not necessarily match with those collected on the farms (Fig 4A and 4B).

Among the clusters with ≥98% similarity there were also groups of isolates originating from the same farm (Fig 4A and 4B), which were confirmed by PFGE (Fig 4C). To better define the strain-relatedness within a single farm and/or a farm compartment (young versus old pigs) we chose three farms with a high number of ESBL-E isolates (B09, B10, B18) for a more detailed analysis (Fig 4D). If more than one morphologically distinct ESBL-E was found on a pig we included both isolates. The findings obtained revealed that despite individual clusters with high (≥ 98%) similarity we mostly detected unrelated *E*. *coli* isolates within one farm (< 95% similarity) (Fig 4D). Furthermore, similarity between isolates from the young and old pigs in B10 and B18 was <95% (Fig 4D). Clusters with ≥95% similarity were usually derived from the same compartment but even within the individual compartment many isolates were unrelated (<95% similarity) (Fig 4D).

### Usage of antibiotics on farms

**Fig 5:**
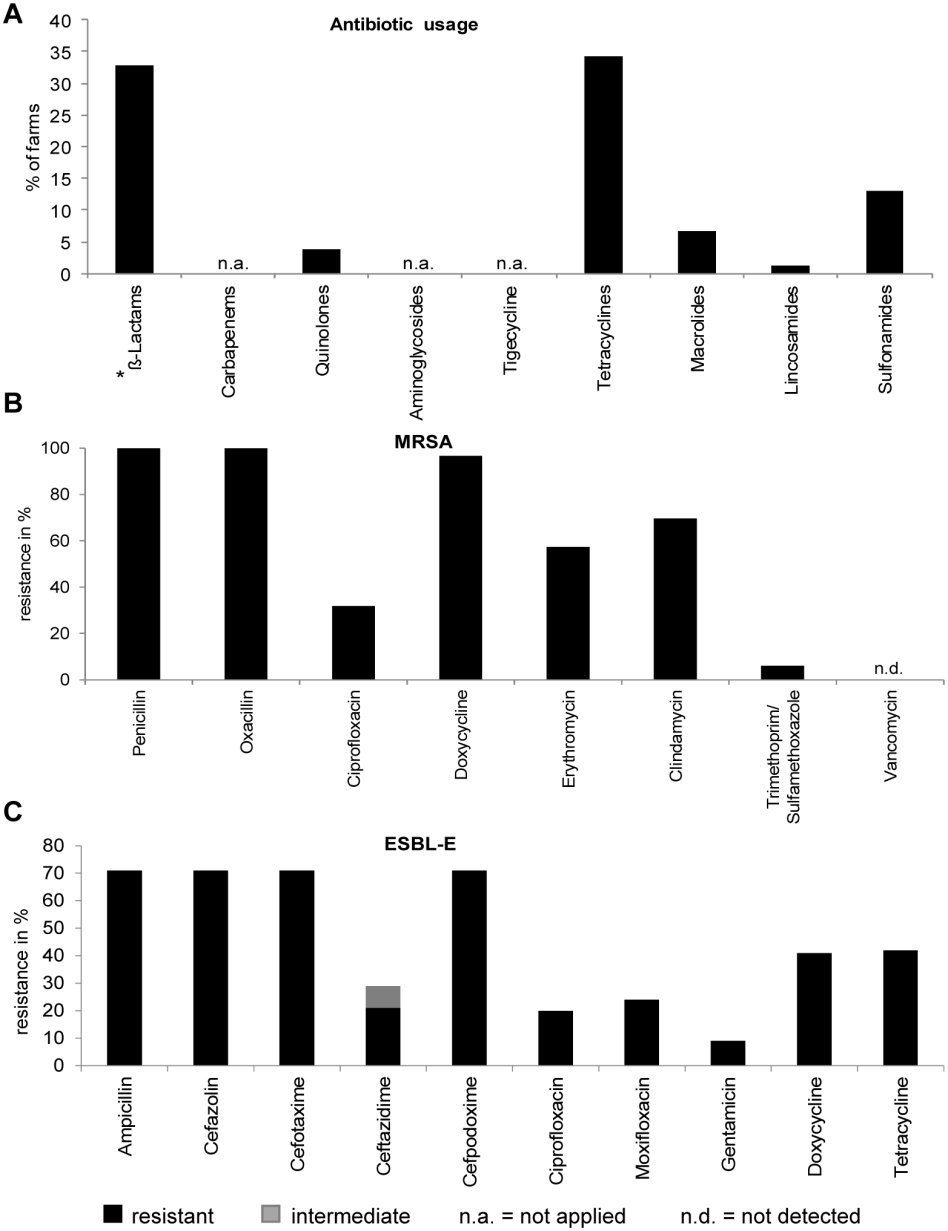
Comparison of antibiotic usage and antibiotic resistance patterns on farms. **A:** Antibiotic substances administered on investigated farms. (*) Betalactams comprise ampicillin, amoxicillin, other penicillins and cephalosporins. Carbapenems, aminoglycosides and tigecycline were not applied (n.a.). **B:** Susceptibility of *spa* typed MRSA isolates obtained on farms given as % resistant. **C:** Susceptibility of tested ESBL-E isolates is provided as % resistant.

The colonization with drug resistant bacteria was compared to the therapeutic usage of antibiotics on the farms. Our analysis showed that betalactam antibiotics and tetracyclines were most frequently administered (24 of 35 (68.6%) and 25 of 35 (71.4%) farms, respectively), thus providing the selective pressure that allows the emergence of MRSA and ESBL-E (Fig 5A). While MRSA and ESBL-E are always resistant to betalactams nearly 100% of MRSA isolates displayed resistance to tetracyclines (Fig 5B) and ESBL-E were resistant to tetracycline in 59.2% (42/71) and to doxycycline in 58.7% (41/71) (Fig 5C). Further analysis showed that on farms that with discontinuation of betalactams (farms B03, B05, B08, B09, B22, B24, B25, B28, B29, B34, B35) the MRSA prevalence was categorized as A or B in 63.6% (7/11) farms while category A or B was found in 58.3% (14/24) of farms that used betalactams. The ESBL-E prevalence lay in category A or B in 36.4% (4/11) of farms that did not administer betalactams and in 50% (12/24 farms that used betalactams. Notably, in 8/10 (80%) farms that suspended tetracycline usage (farms B02, B04, B05, B08, B09, B10, B17, B18, B25, B27) MRSA prevalence was classified as category A or B while in farms administering tetracyclines MRSA category A or B was found on only 13/25 (52%) of farms (see S10 Table).

By contrast, the use of other classes of antibiotics was restricted to a smaller number of farms, e.g. macrolides (5 farms), lincosamides (1 farm), quinolones (3 farms) and sulfonamides (10 farms) (Fig 5A). Nevertheless, antimicrobial susceptibility testing of MRSA revealed resistance to all substances, i.e. erythromycin in 57.7% (113/196) isolates, clindamycin in 76.0% (149/196) isolates, ciprofloxacin in 30.6% (60/196) isolates and to combined trimethoprim/sulfomethoxazole in 5.1% (10/196) isolates (Fig 5B). All MRSA isolates tested were susceptible to vancomycin. Notably, strains resistant to macrolides, lincosamides and quinolones were found independently of the use of these antibiotics on the individual farm.

In ESBL-E we detected resistance to quinolones (ciprofloxacin 28.2% (20/71), moxifloxacin 33% (24/71)) while resistance to carbapenems or combined trimethoprim/sulfomethoxazole was not observed (Fig 5C). Again, quinolone resistance did not correlate with use of these antibiotics on the farms.

## Discussion

Livestock serves as an important reservoir for transferable resistance genes [36, 55, 56]. To our knowledge this study is the first to demonstrate co-colonization with MRSA and ESBL-E on the individual animals (Fig 1). Notably, if on a farm pigs were found to be colonized with MRSA it was likely that ESBL-E were also present on this farm and vice versa, thus, indicating that farm-dependent factors including the amount and class of antibiotics in use foster the selection of drug-resistant pathogens. In support of this hypothesis we further recovered strains resistant to all other antibiotic classes presently in use on the participating pig farms (Fig 5A).

This was further supported by the finding that both MRSA and ESBL-E colonization frequencies varied depending on the pig production level (Fig 2D, S3 and S4 Tables) [57–61]. Higher MRSA colonization in the finishing compartments (Fig 2D, S3 Table) is probably due to previous exposure to antibiotics, which is usually highest at the early stages of breeding (piglets) [62] and transient LA-MRSA colonization. Of note, persistence of colonization in pigs and humans was recently shown to depend on the continuous usage of antibiotics [63–65]. As described in earlier studies both ESBL-E and MRSA detection declined at the ready-to-slaughter stage (Fig 2D, S3 and S4 Tables) [66, 67] which might reflect restricted antibiotic usage at this production level [68, 69].

It seems that antibiotic consumption in the veterinary field is responsible for the spread of drug-resistant bacteria among farm animals [4, 5, 55, 70–73]. In the present study frequent usage of betalactam antibiotics on the participating farms (Fig 5A) was well in line with the presence of bacterial strains resistant to betalactams, i.e. MRSA and ESBL-E, in the pigs. The high usage of tetracyclines might further account for selection of tetracycline-resistant strains (Fig 5A). In our study, the documentation of antibiotic usage on the individual farms revealed that use of tetracyclines rather than that of betalactams might support colonization of LA-MRSA (S10 Table). However, resistance profiles on individual farms did not correlate with other classes of antibiotics in use on the respective farms. Notably, pigs are frequently sold before they enter the next production stage and colonization probably also reflects antibiotic usage on the supplying farm. Furthermore, similarly to humans, colonization with ESBL-E most likely persists for 6 months and longer, even in the absence of antibiotic pressure [74].

The present study confirms earlier results that suggest transmission of MRSA from pigs to humans and vice versa [9, 10, 13, 62, 75, 76]. Moreover, a recent study observed transmission of IncN plasmids harbouring *bla*_CTX-M-1_ between commensal *E*. *coli* of pigs and commensal *E*. *coli* in humans in Denmark [77]. However, there is no evidence for ESBL-E colonization of humans in our farm collective as also reported in [78]. While this could be due to differences in the hygiene regimes employed by farms from different countries, our data further indicate that MRSA transmission might be facilitated by its almost ubiquitous presence in air (Fig 1A, S7 and S8 Tables). Earlier studies support our observations and highlighted stable air as an ideal transmission medium for MRSA [57, 79, 80]. Collectively these data indicate that MRSA contamination of air is more wide-spread than for ESBL-E. Nevertheless, MRSA recovery from impaction samples did not predict MRSA colonization of pigs (Fig 3C). However, we observed MRSA transmission in the abattoirs, which supports earlier findings suggesting that transmission can occur within a short time frame, i.e. in less than two hours [22, 59, 81–83].

In contrast to MRSA, ESBL-E was only rarely detected in air samples (S7 and S8 Tables).

However, ESBL-E detection in impaction samples was always associated with ESBL-E colonization of pigs in the respective farm (Fig 1B). Since humidity is required for persistence of enterobacteriaceae on inanimate surfaces, we further reasoned that the lower presence of ESBL-E in air samples could be due to low humidity in stable air. Well in line with this hypothesis, ESBL-E was detected in 6 of 12 (50%) of air samples in the abattoirs that are kept at higher air humidity (S8 Table) compared to only 6 of 67 (9%) of air samples on farms that have normal environmental humidity (S7 Table). From a technical point of view, future work will have to verify whether impaction is, indeed, superior to impingement in regards to recovery of ESBL-E under normal environmental humidity conditions as suggested for *Salmonella* spp. in [84–86].

Furthermore, ESBL-E transmission was detected in the abattoirs and was higher in abattoir B where pigs were held in a humid environment until slaughtering (S6 and S8 Tables). Although we need to take into consideration that the screening methods used might have failed to detect ESBL-E (and MRSA) in the pigs before delivery to the abattoir, it should be denoted that recent studies have postulated that transmission of *Salmonella* spp. in pigs is fostered by humidity in the abattoir waiting area [84, 85]. Thus, the risk for ESBL-E transmission is probably higher in the abattoirs than on the farms and during transport. Our future studies will, therefore, clarify whether employees working in abattoirs face a higher risk of ESBL-E transmission through pig contact than those working on farms [66].

Altogether, our data allow the conclusion that transmission of MRSA and ESBL-E among pigs during transport and the waiting period at the abattoir might occur with nearly 30% probability. Waiting conditions such as irrigation might influence the likelihood of transmission.

Colonization of livestock with drug resistant bacteria is often considered a risk factor for meat contamination with resistant bacteria [83]. As proposed by earlier studies, colonization of pigs did not result in contamination of carcasses kept in the cool room of the abattoir after slaughter with MRSA and ESBL-E (S6 Table) [25, 87]. This was not surprising because the muscle itself is sterile and the meat production involves strict hygiene measures including mechanical cleansing of the carcass and a series of heat exposures that destroys the microflora. In the present cases, the multi-step procedure included a 60°C hot water treatment and repeated exposures to 2000°C in ovens optimized to reach 100°C within the carcass, i.e. sterilizing conditions. We, therefore, postulate that the risk for contamination of meat is much higher than during processing of the carcasses.

Several studies have identified MRSA lineages that are prototypically found in pigs [22, 44, 57, 59, 83, 88, 89]. Not surprisingly, as seen in our study, these strains are usually resistant to tetracyclines (Fig 5B), another class of antibiotics frequently employed in livestock (Fig 5A) [68]. They further identified the molecular changes that occur in LA-MRSA upon adaptation to the human host [88–91]. Our study highlights the predominance of LA-MRSA associated *spa* types in pigs and humans with direct contacts to pigs and their family members (Fig 3C; S5 Table). Contact to pigs was further associated with increased MRSA colonization. Notably, the MRSA frequency in individuals who had no contact was higher than in the general German population [92, 93] despite the inaccuracy due to the low sample numbers. Altogether, the findings indicated that selective pressure by antibiotics might favour the spread of defined (LA-)MRSA strains among pigs and from pigs to humans or vice versa.

Similarly, specific ESBL resistance genes such as certain CTX-M subgroups have been found with high homogenity within pig herds [49, 50, 66]. It has further been proposed that defined *E*. *coli* strains acquired ESBL resistance genes and then spread among pigs [94]. In the present study molecular typing was performed using a rep-PCR method. The results obtained revealed high diversity of *E*. *coli* ESBL-E isolates (< 95% similarity by DiversiLab typing) when comparing isolates from different farms (Fig 4A). Only small clusters of strains with ≥ 98% similarity revealed a potential spread of strains beyond the individual farm (Fig 4B). What is more, genetic heterogeneity of *E*. *coli* isolates was high, even among strains collected from pigs within the same compartment. This lead to the hypothesis, that ESBL resistance is not transmitted by individual strains, i.e. counterparts of LA-MRSA. It must rather be assumed that selective pressure exerted by antibiotics fosters spread of defined molecular resistance genes and their horizontal transfer within the pre-existing *E*. *coli* population present in the intestinal microflora. Indeed, spread of ESBL-E within a compartment was more complete than that of MRSA, e.g. once ESBL-E was detected on a farm it normally affected nearly all pigs present within the compartment tested (S3 and S4 Tables). By contrast, MRSA colonization rarely affected all pigs within one compartment (S3 Table) although total MRSA colonization was higher than the ESBL-E detection (S4 Table). This suggested that within the compartment ESBL-E is either more rapidly transmitted, ESBL-E colonization is more stable or culture methods used for enrichment were more sensitive for ESBL-E than for LA-MRSA.

Our study results further suggested that, on the contrary to the results obtained on the farms, acquisition of MRSA by the individual pig in the abattoir was more frequent than that of ESBL-E (S6 Table). While antibiotic selection of ESBL-E in the intestine may account for ESBL carriage on the farms, close animal contact in the waiting bay of the abattoir may favour the rapid spread of MRSA. This demonstrates that transmission of resistant bacteria as well as resistance determinants in the pig production chain may vary depending on the environment, antibiotic exposure and bacterial species.

## Supporting Information

S1 Table MRSA and ESBL-E colonization in pigs (sorted by MRSA prevalence on farms). (PDF)

S2 Table MRSA and ESBL-E colonization in pigs (sorted by ESBL-E prevalence on farms). (PDF)

S3 Table MRSA colonization in pigs (sorted by age and production step). (PDF)

S4 Table ESBL-E colonization in pigs (sorted by age and production step). (PDF)

S5 Table MRSA isolates obtained from humans. (PDF)

S6 Table MRSA and ESBL-E colonization of pigs sampled on farms and abattoirs. (PDF)

S7 Table MRSA and ESBL-E detection in farm air. (PDF)

S8 Table MRSA and ESBL-E detection in air on abattoirs. (PDF)

S9 Table MRSA *spa* types on farms (in pigs, humans and air). (PDF)

S10 Table MRSA and ESBL-E colonization in pigs and the use of classes of antibiotics on individual farms. (PDF)
