# Supplementary material for: Analysis of Transmission of MRSA and ESBL-E among Pigs and Farm Personnel
Source: PLoS One. 2015 Sep 30;10(9):e0138173. doi: 10.1371/journal.pone.0138173 (PMC4589321; doi:10.1371/journal.pone.0138173)
Supplement: S2 Table — (PDF) [file pone.0138173.s002.pdf]

**Table S2. MRSA and ESBL-E colonization in pigs (sorted by ESBL-E prevalence on farms).**

| | **Pig - ESBL-E** | | | **Pig - MRSA** | | |
|---|---|---|---|---|---|---|
| **Farms** | negative | positive | % pos | negative | positive | % pos |
| B35 FR | 0 | 7 | 100% | 2 | 8 | 80% |
| B02 FF | 4 | 16 | 80% | 16 | 4 | 20% |
| B31 NF | 3 | 7 | 70% | 6 | 4 | 40% |
| B20 FF | 6 | 14 | 70% | 14 | 6 | 30% |
| B19 FF | 6 | 14 | 70% | 15 | 5 | 25% |
| B30 FR | 3 | 7 | 70% | 8 | 2 | 20% |
| B24 NF | 3 | 7 | 70% | 9 | 1 | 10% |
| B01 FF | 6 | 14 | 70% | 20 | 0 | 0% |
| B22 FF | 4 | 6 | 60% | 10 | 0 | 0% |
| B10 FF | 9 | 11 | 55% | 13 | 6 | 32% |
| B18 FF | 9 | 11 | 55% | 16 | 4 | 20% |
| B33 FR | 5 | 5 | 50% | 9 | 1 | 10% |
| B27 FR | 5 | 5 | 50% | 10 | 0 | 0% |
| B28 FF | 6 | 4 | 40% | 6 | 4 | 40% |
| B09 FF | 12 | 8 | 40% | 12 | 6 | 33% |
| B34 FR | 6 | 4 | 40% | 7 | 3 | 30% |
| B25 FR | 6 | 4 | 40% | 9 | 1 | 10% |
| B06 FR | 13 | 7 | 35% | 15 | 5 | 25% |
| B12 FF | 13 | 5 | 28% | 11 | 9 | 45% |
| B29 FF | 9 | 1 | 10% | 10 | 0 | 0% |
| B13 FF | 16 | 1 | 6% | 18 | 2 | 10% |
| B04 FR | 18 | 1 | 5% | 18 | 2 | 10% |
| B15 FF | 19 | 1 | 5% | 12 | 8 | 40% |
| B14 FF | 19 | 1 | 5% | 15 | 5 | 25% |
| B17 FF | 19 | 1 | 5% | 18 | 2 | 10% |
| B07 FF | 19 | 1 | 5% | 19 | 0 | 0% |
| B11 FF | 20 | 0 | 0% | 10 | 9 | 47% |
| B26 FF | 10 | 0 | 0% | 6 | 4 | 40% |
| B08 FR | 19 | 0 | 0% | 16 | 4 | 20% |
| B16 FF | 20 | 0 | 0% | 16 | 4 | 20% |
| B03 FF | 20 | 0 | 0% | 19 | 2 | 10% |
| B32 FR | 10 | 0 | 0% | 9 | 1 | 10% |
| B21 FF | 20 | 0 | 0% | 20 | 1 | 5% |
| B05 FF | 10 | 0 | 0% | 10 | 0 | 0% |
| B23 FF | 10 | 0 | 0% | 10 | 0 | 0% |

FR = farrowing, NF = nursery, FF = finishing
