# Supplementary material for: Analysis of Transmission of MRSA and ESBL-E among Pigs and Farm Personnel
Source: PLoS One. 2015 Sep 30;10(9):e0138173. doi: 10.1371/journal.pone.0138173 (PMC4589321; doi:10.1371/journal.pone.0138173)
Supplement: S3 Table — (PDF) [file pone.0138173.s003.pdf]

Table S3. MRSA colonization in pigs (sorted by age and production step).

	Pigs		Pig - MRSA		Pig - MRSA	
**Farm**	% pos	comp	young far./ newly we.	farrowing/ nursery	young far./ newly we.	farrowing/ nursery
B35 FR	80%	5	3	5	60%	100%
B34 FR	30%	5	1	2	20%	40%
B06 FR	25%	10	2	3	20%	30%
B08 FR	20%	10	1	3	10%	30%
B30 FR	20%	5	1	1	20%	20%
B04 FR	10%	10	0	2	0%	20%
B25 FR	10%	5	0	1	0%	20%
B32 FR	10%	5	0	1	0%	20%
B33 FR	10%	5	0	1	0%	20%
B27 FR	0%	5	0	0	0%	0%
B31 NF	40%	5	4	0	80%	0%
B24 NF	10%	5	0	1	0%	20%
**Farm**	% pos	comp	early finisher	finisher	early finisher	finisher
B11 FF	47%	10	8	1	80%	10%
B12 FF	45%	10	5	4	50%	40%
B15 FF	40%	10	8	0	80%	0%
B26 FF	40%	5	3	1	60%	20%
B28 FF	40%	5	4	0	80%	0%
B09 FF	33%	10	6	0	60%	0%
B10 FF	32%	10	6	0	60%	0%
B20 FF	30%	10	4	2	40%	20%
B14 FF	25%	10	2	3	20%	30%
B19 FF	25%	10	5	0	50%	0%
B02 FF	20%	10	3	1	30%	10%
B16 FF	20%	10	4	0	40%	0%
B18 FF	20%	10	3	1	30%	10%
B03 FF	10%	10	1	1	10%	10%
B13 FF	10%	10	1	1	10%	10%
B17 FF	10%	10	0	2	0%	20%
B21 FF	5%	10	1	0	10%	0%
B01 FF	0%	10	0	0	0%	0%
B05 FF	0%	10	0	0	0%	0%
B07 FF	0%	10	0	0	0%	0%
B22 FF	0%	5	0	0	0%	0%
B23 FF	0%	5	0	0	0%	0%
B29 FF	0%	5	0	0	0%	0%

comp = per compartment, FR = farrowing, NF = nursery, FF = finishing
young far. = young farrowing piglet, newly we. = newly weaned piglet
