# Supplementary material for: Analysis of Transmission of MRSA and ESBL-E among Pigs and Farm Personnel
Source: PLoS One. 2015 Sep 30;10(9):e0138173. doi: 10.1371/journal.pone.0138173 (PMC4589321; doi:10.1371/journal.pone.0138173)
Supplement: S4 Table — (PDF) [file pone.0138173.s004.pdf]

Table S4. ESBL-E colonization in pigs (sorted by age and production step).

	**Pig**		**Pig - ESBL-E**		**Pig - ESBL-E**	
**Farm**	% pos	comp	young far./ newly we.	farrowing/ nursery	young far./ newly we.	farrowing/ nursery
B35 FR	100%	5	3	4	60%	80%
B30 FR	70%	5	3	4	60%	80%
B33 FR	50%	5	4	1	80%	20%
B27 FR	50%	5	1	4	20%	80%
B34 FR	40%	5	2	2	40%	40%
B25 FR	40%	5	2	2	40%	40%
B06 FR	35%	10	7	0	70%	0%
B04 FR	5%	10	1	0	10%	0%
B08 FR	0%	10	0	0	0%	0%
B32 FR	0%	5	0	0	0%	0%
B31 NF	70%	5	3	4	60%	80%
B24 NF	70%	5	4	3	80%	60%
**Farm**	% pos	comp	early finisher	finisher	early finisher	finisher
B02 FF	80%	10	10	6	100%	60%
B20 FF	70%	10	8	6	80%	60%
B19 FF	70%	10	10	4	100%	40%
B01 FF	70%	10	4	10	40%	100%
B22 FF	60%	5	5	1	100%	20%
B10 FF	55%	10	10	1	100%	10%
B18 FF	55%	10	6	5	60%	50%
B28 FF	40%	5	4	0	80%	0%
B09 FF	40%	10	8	0	80%	0%
B12 FF	28%	10	2	3	20%	30%
B29 FF	10%	5	1	0	20%	0%
B13 FF	6%	10	1	0	10%	0%
B15 FF	5%	10	1	0	10%	0%
B14 FF	5%	10	1	0	10%	0%
B17 FF	5%	10	0	1	0%	10%
B07 FF	5%	10	0	1	0%	10%
B11 FF	0%	10	0	0	0%	0%
B26 FF	0%	5	0	0	0%	0%
B16 FF	0%	10	0	0	0%	0%
B03 FF	0%	10	0	0	0%	0%
B21 FF	0%	10	0	0	0%	0%
B05 FF	0%	10	0	0	0%	0%
B23 FF	0%	5	0	0	0%	0%

comp = per compartment, FR = farrowing, NF = nursery, FF = finishing
young far. = early farrowing piglet, newly we. = newly weaned piglet
