# Supplementary material for: Analysis of Transmission of MRSA and ESBL-E among Pigs and Farm Personnel
Source: PLoS One. 2015 Sep 30;10(9):e0138173. doi: 10.1371/journal.pone.0138173 (PMC4589321; doi:10.1371/journal.pone.0138173)
Supplement: S5 Table — (PDF) [file pone.0138173.s005.pdf]

Table S5. MRSA isolates obtained from humans.

	MRSA				
	**Human samples**		**Positive**		
**Farms**	negative	positive	Farmer	Staff	Family
B35 FR	0	2	0	2	0
B11 FF	0	2	1	0	1
B12 FF	0	2	1	0	1
B15 FF	1	1	1	0	0
B26 FF	1	1	1	0	0
B28 FF	2	2	0	1	1
B31 NF	0	2	1	0	1
B09 FF	2	2	1	0	1
B10 FF	1	1	1	0	0
B20 FF	3	1	1	0	0
B34 FR	0	1	0	1	0
B06 FR	1	2	1	1	0
B14 FF	2	3	1	1	1
B19 FF	2	1	0	0	1
B02 FF	1	0	0	0	0
B08 FR	4	0	0	0	0
B16 FF	1	2	0	2	0
B18 FF	2	0	0	0	0
B30 FR	2	3	1	0	2
B03 FF	1	0	0	0	0
B04 FR	1	2	1	0	1
B13 FF	1	1	1	0	0
B17 FF	2	0	0	0	0
B24 NF	1	0	0	0	0
B25 FR	1	1	1	0	0
B32 FR	0	3	1	1	1
B33 FR	0	1	0	1	0
B21 FF	1	2	1	1	0
B01 FF	1	0	0	0	0
B05 FF	1	1	1	0	0
B07 FF	3	0	0	0	0
B22 FF	0	2	1	0	1
B23 FF	3	0	0	0	0
B27 FR	3	0	0	0	0
B29 FF	0	1	1	0	0

FR = farrowing, NF = nursery, FF = finishing
