# Supplementary material for: Analysis of Transmission of MRSA and ESBL-E among Pigs and Farm Personnel
Source: PLoS One. 2015 Sep 30;10(9):e0138173. doi: 10.1371/journal.pone.0138173 (PMC4589321; doi:10.1371/journal.pone.0138173)
Supplement: S6 Table — (PDF) [file pone.0138173.s006.pdf]

Table S6. MRSA und ESBL-E colonization of pigs sampled on farms and abattoirs.

	Farms						Abattoirs					
	ESBL-E			MRSA			ESBL-E			MRSA		
**Farms**	negative	positive	% pos	% pos	negative	positive	t1	t2	t3	t1	t2	t3
B02 FF	4	16	80%	20%	16	4	2	1	0	0	0	0
B19 FF	6	14	70%	25%	15	5	1	2	0	0	0	0
B18 FF	9	11	55%	20%	16	4	2	2	0	0	0	0
B15 FF	19	1	5%	40%	12	8	0	0	0	0	1	0
B14 FF	19	1	5%	25%	15	5	0	2	0	2	3	0
B16 FF	20	0	0%	20%	16	4	0	3	0	2	1	0
B13 FF	16	1	6%	10%	18	2	0	2	0	2	3	0
B17 FF	19	1	5%	10%	18	2	1	0	0	3	1	0
B29 FF	9	1	10%	0%	10	0	0	0	0	0	2	0
B03 FF	20	0	0%	10%	19	2	0	0	0	0	2	0
B07 FF	19	1	5%	0%	19	0	1	1	0	1	3	0
B21 FF	20	0	0%	5%	20	1	0	0	0	1	3	0
B23 FF	10	0	0%	0%	10	0	0	0	0	2	2	0

t = time point, FF = finishing
