# Supplementary material for: Analysis of Transmission of MRSA and ESBL-E among Pigs and Farm Personnel
Source: PLoS One. 2015 Sep 30;10(9):e0138173. doi: 10.1371/journal.pone.0138173 (PMC4589321; doi:10.1371/journal.pone.0138173)
Supplement: S7 Table — (PDF) [file pone.0138173.s007.pdf]

Table S7. MRSA und ESBL-E detection in farm air.

Air samples			
**MRSA**		**ESBL-E**	
negative	positive	negative	positive
0	2	2	0
0	2	2	0
0	2	2	0
0	3	1	1
0	2	0	1
0	2	2	0
1	1	2	0
1	1	1	1
0	3	2	0
0	2	1	1
0	2	2	0
0	2	1	1
0	2	2	0
1	1	2	0
0	2	2	0
0	1	2	0
0	2	2	0
0	2	1	1
1	1	2	0
0	2	2	0
1	1	2	0
0	2	2	0
0	2	2	0
0	3	2	0
0	2	2	0
0	2	2	0
1	1	2	0
1	1	2	0
0	2	1	0
0	1	2	0
1	1	2	0
0	2	2	0
0	2	2	0
2	0	1	0
0	1	2	0
