# Supplementary material for: Analysis of Transmission of MRSA and ESBL-E among Pigs and Farm Personnel
Source: PLoS One. 2015 Sep 30;10(9):e0138173. doi: 10.1371/journal.pone.0138173 (PMC4589321; doi:10.1371/journal.pone.0138173)
Supplement: S8 Table — (PDF) [file pone.0138173.s008.pdf]

Table S8. MRSA und ESBL-E detection in air on abattoirs.

**Air samples**			
Abattoir	MRSA / ESBL-E	t2	t3
Abattoir A	MRSA	+	-
		+	-
		+	-
		+	-
		-	-
		+	-
	ESBL-E	-	-
		-	-
		+	-
		+	-
		+	-
Abattoir B	MRSA	+	n.d.
		+	n.d.
		+	n.d.
		+	n.d.
		+	n.d.
		+	n.d.
		+	n.d.
		+	n.d.
	ESBL-E	+	n.d.
		-	n.d.
		+	n.d.
		+	n.d.
		-	n.d.
		-	n.d.
		-	n.d.

t = time point, n.d. = not determined
