# Supplementary material for: Analysis of Transmission of MRSA and ESBL-E among Pigs and Farm Personnel
Source: PLoS One. 2015 Sep 30;10(9):e0138173. doi: 10.1371/journal.pone.0138173 (PMC4589321; doi:10.1371/journal.pone.0138173)
Supplement: S9 Table — (PDF) [file pone.0138173.s009.pdf]

Table S9. MRSA *spa* types on farms (in pigs, humans and air).

	**Pig**							**Human**					**Air**								
farms/ ***spa* types**	**t011**	**t034**	**t108**	**t1255**	**t1451**	**t1456**	**t2011**	**t011**	**t034**	**t1255**	**t2011**	**t2330**	**t011**	**t034**	**t108**	**t1255**	**t1451**	**t2011**	**t2123**	**t898**	**∑**
B14	**2**	**1**						**2**		**1**			**2**								**8**
B11	**1**				**2**			**2**					**1**				**1**				**7**
B12	**3**							**2**					**2**								**7**
B35	**2**							**2**					**2**								**6**
B32	**1**							**3**					**2**								**6**
B30	**1**			**1**				**1**		**2**						**1**					**6**
B06	**1**						**1**	**2**					**1**					**1**			**6**
B16			**2**					**1**	**1**						**2**						**6**
B13	**2**							**1**					**2**								**5**
B10	**1**								**1**				**1**	**1**	**1**						**5**
B26	**2**							**1**					**1**	**1**							**5**
B34	**2**							**1**					**2**								**5**
B15		**1**						**1**						**3**							**5**
B28	**1**							**1**	**1**				**2**								**5**
B09		**2**						**1**	**1**					**1**							**5**
B20	**2**											**1**	**1**		**1**						**5**
B02	**2**												**1**		**1**						**4**
B19	**1**		**1**								**1**		**1**								**4**
B31		**1**						**1**	**1**				**1**								**4**
B24	**1**												**2**	**1**							**4**
B25		**1**						**1**					**1**	**1**							**4**
B03		**2**												**2**							**4**
B21	**1**							**2**					**1**								**4**
B04		**1**						**1**	**1**					**1**							**4**
B22								**2**					**1**	**1**							**4**

B08	**2**												**1**								**3**
B33	**1**							**1**					**1**								**3**
B17						**1**														**2**	**3**
B18	**1**																		**1**		**2**
B01													**1**		**1**						**2**
B29								**1**					**1**								**2**
B23													**1**	**1**							**2**
B05								**1**					**1**								**2**
B07													**1**								**1**
**∑**	30	9	3	1	2	1	1	31	6	3	1	1	34	13	6	1	1	1	1	2	
in %	64%	19%	6%	2%	4%	2%	2%	74%	14%	7%	2%	2%	58%	22%	10%	2%	2%	2%	2%	3%	
