# Supplementary material for: Analysis of Transmission of MRSA and ESBL-E among Pigs and Farm Personnel
Source: PLoS One. 2015 Sep 30;10(9):e0138173. doi: 10.1371/journal.pone.0138173 (PMC4589321; doi:10.1371/journal.pone.0138173)
Supplement: S10 Table — (PDF) [file pone.0138173.s010.pdf]

Table S10. MRSA and ESBL-E colonization in pigs and the use of classes of antibiotics on individual farms.

	**Pig - MRSA**		**Pig - ESBL-E**		**antibiotics**
**Farms**	% positive	category	% positive	category	classes
B01 FF	0%	A	70%	D	Betalactams, Tetracyclines
B02 FF	20%	B	80%	D	Betalactams
B03 FF	10%	B	0%	A	Tetracyclines
B04 FR	10%	B	5%	B	Betalactams
B05 FF	0%	A	0%	A	Chloramphenicol
B06 FR	25%	C	35%	C	Betalactams, Tetracyclines
B07 FF	0%	A	5%	B	Betalactams, Tetracyclines
B08 FR	20%	B	0%	A	
B09 FF	33%	C	40%	C	Tetracyclines, Sulfonamides
B10 FF	32%	C	55%	D	Betalactams
B11 FF	47%	C	0%	A	Betalactams, Tetracyclines, Sulfonamides
B12 FF	45%	C	28%	C	Betalactams, Tetracyclines, Sulfonamides
B13 FF	10%	B	6%	B	Betalactams, Tetracyclines, Colistin
B14 FF	25%	C	5%	B	Betalactams, Tetracyclines, Macrolides, Colistin
B15 FF	40%	C	5%	B	Betalactams, Tetracyclines
B16 FF	20%	B	0%	A	Betalactams, Tetracyclines
B17 FF	10%	B	5%	B	Betalactams
B18 FF	20%	B	55%	D	Betalactams
B19 FF	25%	C	70%	D	Betalactams
B20 FF	30%	C	70%	D	Betalactams, Tetracyclines, Lincosamides
B21 FF	5%	B	0%	A	Betalactams, Tetracyclines, Sulfonamides
B22 FF	0%	A	60%	D	Tetracyclines, Sulfonamides
B23 FF	0%	A	0%	A	Betalactams, Tetracyclines
B24 NF	10%	B	70%	D	Tetracyclines, Sulfonamides
B25 FR	10%	B	40%	C	

B26 FF	40%	C	0%	A	Betalactams, Tetracyclines, Sulfonamides
B27 FR	0%	A	50%	C	Betalactams, Quinolones
B28 FF	40%	C	40%	C	Tetracyclines
B29 FF	0%	A	10%	B	Tetracyclines, Macrolides
B30 FR	20%	B	70%	D	Betalactams, Quinolones, Tetracyclines
B31 NF	40%	C	70%	D	Betalactams, Tetracyclines, Colistin
B32 FR	10%	B	0%	A	Betalactams, Quinolones, Tetracyclines, Macrolides, Sulfonamides
B33 FR	10%	B	50%	C	Betalactams, Tetracyclines, Sulfonamides
B34 FR	30%	C	40%	C	Tetracyclines, Macrolides
B35 FR	80%	D	100%	D	Tetracyclines, Macrolides, Colistin

FR = farrowing, NF = nursery, FF = finishing
